# Gender differences in roles of health behavior between marital status and oral health

**DOI:** 10.1111/ggi.70170

**Published:** 2025-09-11

**Authors:** Farzana Sharmin, Yusuke Matsuyama, Shiho Kino, Sakura Kiuchi, Katsunori Kondo, Jun Aida

**Affiliations:** ^1^ Department of Dental Public Health Graduate School of Medical and Dental Sciences, Institute of Science Tokyo Tokyo Japan; ^2^ Department of Preventive Oral Health Care Sciences Graduate School of Medical and Dental Sciences, Institute of Science Tokyo Tokyo Japan; ^3^ Frontier Research Institute for Interdisciplinary Science, Tohoku University Sendai Japan; ^4^ Department of International and Community Oral Health Tohoku University Graduate School of Dentistry Sendai Miyagi Japan; ^5^ Department of Social Preventive Medical Sciences Chiba University, Center for Preventive Medical Sciences Chiba Japan

**Keywords:** gender difference, marital status, mediation analysis, number of teeth, oral health behavior

## Abstract

**Aim:**

Marital status is a form of social network and source of social support, and it is beneficial to oral health by enhancing health behaviors. We examined the mediating effects of oral health behaviors on the association of marital status with the number of teeth.

**Methods:**

This cross‐sectional study used data from the Japan Gerontological Evaluation Study 2022, targeting individuals aged ≥65 years. Marital status was the exposure, and the number of teeth was the outcome. Mediation effects of dental treatment, dental checkup, tooth brushing, alcohol drinking, and smoking were evaluated using the Karlson–Holm–Breen (KHB) method.

**Results:**

Of the 21 745 participants, the mean number of teeth was higher in participants having a spouse (men: 18.49 [standard deviation, SD = 10.00], women: 20.08 [SD = 8.88]) than in those without a spouse (men: 16.34 [SD = 10.34], women: 17.22 [SD = 10.35]). Having a spouse was associated with a higher number of teeth, yielding coefficients of 0.76 (95% confidence interval [CI] = 0.26; 1.25) for men and 0.67 (95% CI = 0.31; 1.03) for women, after adjusting for confounders. The coefficients for the association through oral health behaviors were 0.66 (95% CI = 0.51; 0.81) for men and 0.10 (95% CI = 0.02; 0.19) for women, explaining 46.62% of the association in men and 13.68% in women.

**Conclusions:**

Being married at an older age was associated with having a higher number of teeth, which was mediated by better oral health behaviors. These behaviors had a greater impact on men, who generally had poorer behaviors compared with women. **Geriatr Gerontol Int 2025; 25: 1397–1403**.

## Graphical abstract

The association between marital status and the number of remaining teeth was examined in an older Japanese population, and the mediating role of oral health behaviors was analyzed, considering gender differences in behavioral pathways.

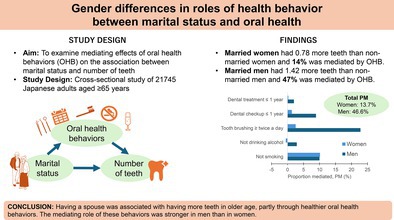

## Introduction

Oral health problems are highly prevalent in old age, alongside other physical and mental health conditions.^1^, ^2^, ^3^ Oral health is a critical aspect of overall well‐being, influencing functions such as eating, speaking, and maintaining quality of life.^4^ Tooth loss is a common issue, particularly among older adults,^2^ and has a significant impact on both physical and psychological well‐being. It impairs essential oral functions such as chewing and speaking, potentially causing nutritional deficiencies.^5^ Psychologically, tooth loss can lead to mental health issues, with concerns about appearance and social interaction causing negative emotions and increasing social isolation.^6^, ^7^

Social relationships and networks provide emotional, informational, and instrumental support that enhances physical and mental health.^8^ These relationships play a pivotal role in shaping an individual's health outcomes and behaviors.^9^, ^10^ Marriage, as a social institution, significantly influences health outcomes by offering emotional support and promoting health‐related behaviors between spouses.^11^, ^12^, ^13^, ^14^ Marital status is a significant determinant of oral health, as it can shape oral health behaviors through partner interactions and shared household routines. For example, married individuals in long‐term relationships with children aged 5 to 7 years mutually influence each other's oral health behaviors.^15^ A previous study found that non‐married men aged 40 and above were significantly less likely to seek dental treatment than married men, while there was no significant difference between married and non‐married women.^16^

However, the association between marital status and the number of remaining teeth remains unquantified, and research on gender differences and the older population is limited. As the population ages, bereavement becomes more common, and the number of individuals who have never married has also increased in recent years.^17^ Therefore, this study investigates how marital status, a key social network factor, influences the number of teeth in older adults, focusing on the mediating role of oral health behaviors and emphasizing gender differences.

## Materials and methods

### Study design and population

This cross‐sectional study used data from the Japan Gerontological Evaluation Study (JAGES),^18^, ^19^ a prospective cohort study that collaborates with municipal governments targeting independent older adults aged ≥65 years in Japan to promote gerontological and social epidemiological research.^20^, ^21^ In 2022, 75 municipalities participated in the survey. A self‐reported questionnaire was posted to 338 742 participants, who were chosen basically through random sampling. The questionnaire was returned voluntarily and considered as written informed consent. The number of responses collected was 227 731, with a response rate of 67.2%. The questionnaire included core, sub‐core, and non‐core components. This study included one‐eighth of all participants randomly assigned to the non‐core version questionnaire regarding the current number of teeth and oral health behavior, which 24 093 people completed. We excluded 2348 participants who either indicated a requirement for daily life care or assistance, or did not provide a response to this question or to the question about gender. Thus, the final number of participants was 21 745.

### Exposure variable

Marital status was used as an exposure variable. The respondents answered, “Which of the following applies to your marital status?” Responses included “Have a spouse,” “Bereavement,” “Separated,” “Never married,” or “Other.” Marital status was categorized as having a spouse (having a spouse) or not having a spouse (bereavement, separated, never married, or other). In addition to this binary variable, a sensitivity analysis was conducted to explore the association between detailed marital status categories (having a spouse, bereavement, separated, never married, and other) and the number of teeth.

### Outcome variable

The number of teeth, as a continuous variable, was the outcome variable. The question “How many natural teeth do you have?” was used to determine the number of natural teeth. Previous studies have shown that self‐reported number of teeth is a reasonably reliable method in large population‐based research.^7^, ^22^ Also, the World Health Organization (WHO) supports the use of simplified structured questionnaires for collecting self‐assessed numbers of teeth and other oral health data in adults.^23^

### Mediators

The mediators in this study included dental treatment, dental checkups, tooth brushing frequency, alcohol consumption, and smoking. While dental treatment, dental checkups, and tooth brushing are directly related to oral health, alcohol consumption and smoking are also important owing to their substantial impact on oral health outcomes.^24^, ^25^ For example, smoking is a significant risk factor for periodontal disease,^26^, ^27^, ^28^ and alcohol consumption can influence oral health through various mechanisms (e.g., dry mouth, increased risk of oral cancer).^29^ Therefore, we have included smoking and alcohol consumption under the category of oral health behaviors. A detailed explanation of these variables is shown in the Supplementary Method.

### Confounders

We adjusted for confounders of demographic (age, gender), socioeconomic (education, annual household income including pension), and physical and mental health status (diabetes mellitus, depressive symptoms assessed with the 15‐item Geriatric Depression Scale [GDS], and instrumental activities of daily living [IADL]). A detailed explanation of these variables is shown in the Supplementary Method.

### Statistical analysis

**Figure 1:**
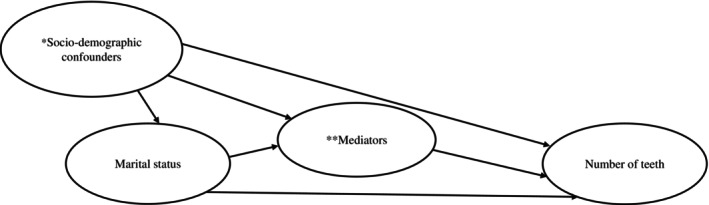
Directed acyclic graph for the mediation analysis. *Sociodemographic covariates: age, sex, education level, income, instrumental activity of daily living (IADL), GDS (geriatric depression scale), and diabetes mellitus. **Mediators: dental treatment, dental checkup, tooth brushing, alcohol drinking and smoking.

First, we descriptively summarized the participants' characteristics stratified by gender. Second, multivariable linear regression analysis was employed to investigate the association between the presence of a spouse and the number of teeth. Univariate models and a fully adjusted model controlling for all mediators and confounders were estimated to assess their associations with the number of teeth. A sensitivity analysis was conducted by using detailed marital status categories (having a spouse, bereavement, separated, never married, and other). Third, to summarize the mediation effect of the mediators, a mediation analysis with the product method was conducted using the Karlson–Holm–Breen (KHB) method,^30^ estimating the mediated proportion using multiple mediators after adjusting for confounders (Fig. 1).

Of the 21 745 participants, 13 253 (60.9%) were complete cases. The proportion of missing information ranged from 0% for age and gender to 14.5% for depressive symptoms (Table S1). To address the missing values, we employed multiple imputation with chained equations, created 20 complete datasets, and combined the results using Rubin's combination method.^31^ All data analyses were stratified by gender and conducted using STATA 17 software (Stata Corporation, College Station, TX, USA).

## Results

Table 1 presents the descriptive characteristics of the 21 745 participants (men, 47.7%; women, 52.3%). The proportion of men with a spouse (83.3%) was higher than that of women with a spouse (62.3%). The mean number of teeth was higher in participants having a spouse (men: 18.49 [standard deviation, SD = 10.00]; women: 20.08 [SD = 8.88]) than in those without a spouse (men: 16.34 [SD = 10.34]; women: 17.22 [SD = 10.35]). Regarding oral health behaviors, 59.8% of men with a spouse and 65.2% of women with a spouse had received dental treatment within the previous year. Tooth brushing was practiced twice or more a day by 66.5% of men with a spouse and by 87.5% of women with a spouse. The proportion of smokers was higher among individuals who did not have a spouse, with 21.3% of men and 4.7% of women being smokers, compared with 16.1% of men and 3.3% of women who had a spouse.

**Table 1:** Gender‐stratified descriptive statistics by marital status after multiple imputation (*n* = 21 745)

		Men (*n* = 10 374)			Women (*n* = 11 371)		
			Not having a spouse	Having a spouse		Not having a spouse	Having a spouse
		Total	*n* = 1725 (16.7%)	*n* = 8650 (83.3%)	Total	*n* = 4288 (37.7%)	*n* = 7083 (62.3%)
		*n* (%)	%	%	*n* (%)	%	%
Number of teeth, mean (SD)		18.14 (9.46)	16.34 (10.34)	18.49 (10.00)	19.01 (8.96)	17.22 (10.35)	20.08 (8.88)
Age	65–69	2382 (23.0)	24.5	22.7	2656 (23.4)	13.9	29.1
	70–74	3086 (29.7)	28.7	29.9	3411 (30.0)	23.4	34.0
	75–79	2400 (23.1)	19.4	23.9	2596 (22.8)	24.1	22.1
	80–84	1637 (15.8)	16.1	15.7	1828 (16.1)	23.0	11.9
	≥85	869 (8.4)	11.3	7.80	881 (7.70)	15.6	2.90
Education level	≤ 9 years	2005 (19.3)	24.5	18.3	2633 (23.2)	30.0	19.0
	10–12 years	4286 (41.3)	40.1	41.5	5302 (46.6)	45.0	47.6
	≥ 13 years	4083 (39.4)	35.4	40.2	3436 (30.2)	25.0	33.4
Income (million JPY)	Low (<2.0)	4790 (46.2)	53.1	44.8	6038 (53.1)	63.6	46.7
	Mid (2.0–3.9)	4202 (40.5)	35.7	41.5	4133 (36.3)	28.6	41.1
	High (≥4.0)	1383 (13.3)	11.2	13.7	1200 (10.6)	7.80	12.2
IADL	Limitation	2556 (24.6)	17.9	25.9	4330 (38.1)	33.8	40.7
	No limitation	7818 (75.4)	82.1	74.1	7041 (61.9)	66.2	59.3
GDS	Non	7929 (76.4)	62.7	79.2	8589 (75.5)	72.5	77.4
	Mild	1959 (18.9)	26.9	17.3	2213 (19.5)	21.2	18.4
	Severe	485 (4.70)	10.4	3.50	569 (5.0)	6.30	4.20
Diabetes mellitus	Not present	8405 (81.0)	80.3	81.2	10 169 (89.4)	88.9	89.7
	Present	1969 (19.0)	19.7	18.8	1202 (10.6)	11.1	10.3
Dental treatment	More than 1 year ago	4317 (41.6)	48.8	40.2	4110 (36.1)	38.3	34.8
	Within 1 year	6057 (58.4)	51.2	59.8	7261 (63.9)	61.7	65.2
Dental checkup	More than 1 year ago	5057 (48.7)	56.1	47.3	4576 (40.2)	42.6	38.8
	Within 1 year	5317 (51.3)	43.9	52.7	6795 (59.8)	57.4	61.2
Tooth brushing	Once a day or less	3691 (35.6)	46.1	33.5	1586 (13.94)	16.3	12.5
	Twice a day or more	6684 (64.4)	53.9	66.5	9785 (86.06)	83.7	87.5
Alcohol drinking*	Yes	6359 (61.3)	56.5	62.2	2591 (22.8)	20.7	24.1
	No	4015 (38.7)	43.5	37.8	8780 (77.2)	79.3	75.9
Smoking**	Yes	1761 (17.0)	21.3	16.1	436 (3.80)	4.7	3.3
	No	8613 (83.0)	78.7	83.9	10 935 (96.2)	95.3	96.7

*Note*: Descriptive statistics obtained after multiple imputation.

Abbreviation: GDS, Geriatric Depression Scale; IADL, instrumental activities of daily living; SD, standard deviation.

* Alcohol drinking: Yes (currently drinking); No (stopped within 5 years and not drinking now, stopped drinking >5 years ago and not drinking now, never drank to begin with).

** Smoking: Yes (I smoke almost every day, I smoke occasionally); No (quit within 5 years and do not smoke now, quit >5 years ago and do not smoke now, never smoked before).

Table 2 shows the results of the linear regression analysis of the association between the presence of a spouse and the number of teeth. In the univariate model, the number of remaining teeth was higher for both men (2.14 [95% confidence interval, CI = 1.61; 2.68]) and women (2.86 [95% CI = 2.48; 3.23]) with a spouse than for those without a spouse. In the fully adjusted model, both men and women having a spouse had a significantly higher number of remaining teeth compared with participants not having a spouse (men: 0.76 [95% CI = 0.24; 1.27]; women: 0.67 [95% CI = 0.31; 1.04]).

**Table 2:** Gender‐stratified linear regression analysis of the association of marital status and mediators with number of teeth after multiple imputation (*n* = 21 745)

		Number of teeth, mean (SD)	Univariable model	Fully adjusted model
			coefficient (95% CI)	coefficient (95% CI)
Men (*n* = 10 374)				
Marital status	Not having a spouse	16.34 (10.34)	Reference	Reference
	Having a spouse	18.49 (10.00)	2.14 (1.61; 2.68)	0.76 (0.24; 1.27)
Dental treatment	More than 1 year ago	16.54 (10.62)	Reference	Reference
	Within 1 year	19.26 (9.30)	2.71 (2.32; 3.10)	0.44 (−0.02; 0.90)
Dental checkup	More than 1 year ago	16.44 (10.53)	Reference	Reference
	Within 1 year	19.73 (9.15)	3.29 (2.91; 3.66)	1.98 (1.53; 2.43)
Tooth brushing	Once a day or less	15.09 (11.05)	Reference	Reference
	Twice a day or more	19.81 (8.90)	4.72 (4.30; 5.13)	3.23 (2.84; 3.63)
Alcohol drinking*	Yes	18.93 (9.78)	Reference	Reference
	No	16.86 (10.21)	−2.06 (−2.46; −1.66)	−1.16 (−1.53; −0.80)
Smoking**	Yes	15.34 (10.58)	Reference	Reference
	No	18.70 (9.63)	3.35 (2.82; 3.88)	3.39 (2.89; 3.89)
Women (*n* = 11 371)				
Marital status	Not having a spouse	17.22 (10.35)	Reference	Reference
	Having a spouse	20.08 (8.88)	2.86 (2.48; 3.23)	0.67 (0.31; 1.04)
Dental treatment	More than 1 year ago	17.21 (10.82)	Reference	Reference
	Within 1 year	20.02 (8.51)	2.80 (2.42; 3.19)	0.24 (−0.22; 0.71)
Dental checkup	More than 1 year ago	16.91 (10.56)	Reference	Reference
	Within 1 year	20.42 (8.35)	3.50 (3.14; 3.87)	2.19 (1.73; 2.64)
Tooth brushing	Once a day or less	14.23 (10.88)	Reference	Reference
	Twice a day or more	19.78 (8.98)	5.54 (4.98; 6.11)	3.55 (3.02; 4.08)
Alcohol drinking*	Yes	20.08 (8.87)	Reference	Reference
	No	18.69 (9.67)	−1.38 (−1.78; −0.98)	−0.29 (−0.67; −0.08)
Smoking**	Yes	15.81 (10.10)	Reference	Reference
	No	19.13 (9.53)	3.32 (2.35; 4.29)	3.3 (2.42; 4.26)

*Note*: Fully adjusted model included all listed variables simultaneously and adjusted for the following confounders: age, gender, education level, annual household income, instrumental activities of daily living (IADL), Geriatric Depression Scale (GDS), diabetes mellitus.

Abbreviation: CI, confidence interval; GDS, Geriatric Depression Scale; IADL, instrumental activities of daily living; SD, standard deviation.

* Alcohol drinking: Yes (currently drinking); No (stopped within 5 years and not drinking now, stopped drinking >5 years ago and not drinking now, never drank to begin with).

** Smoking: Yes (I smoke almost every day, I smoke occasionally); No (quit within 5 years and do not smoke now, quit >5 years ago and do not smoke now, never smoked before).

Table 3 shows the mediation effects of oral health behaviors on the association between the presence of a spouse and the number of teeth. The total effects suggested a positive association between marital status and the number of teeth in both men and women. For men, the coefficient was 1.42 (95% CI = 0.93; 1.91) and for women, the coefficient was 0.78 (95% CI = 0.42; 1.14). The direct effects were 0.76 (95% CI = 0.26; 1.25) for men and 0.67 (95% CI = 0.31; 1.03) for women. The indirect effect through the mediators was 0.66 (95% CI = 0.51; 0.81) and 0.10 (95% CI = 0.02; 0.19) for men and women, respectively. Mediators accounted for 46.62% of the association in men and for 13.68% in women.

**Table 3:** Total, direct, and indirect effects of marital status on the number of teeth by the Karlson–Holm–Breen (KHB) method with multiple imputation (*n* = 21 745)

	Coefficient (95% CI)
Men (*n* = 10 374)	
Total effect	1.42 (0.93; 1.91)
Direct effect	0.76 (0.26; 1.25)
Indirect effect	0.66 (0.51; 0.81)
Proportion mediated (%)	46.62
Women (*n* = 11 371)	
Total effect	0.78 (0.42; 1.14)
Direct effect	0.67 (0.31; 1.03)
Indirect effect	0.10 (0.02; 0.19)
Proportion mediated (%)	13.68

*Note*: Marital status as exposure, dental treatment, dental checkup, tooth brushing frequency, alcohol drinking, and smoking were used as mediators. Age, gender, education level, annual household income, instrumental activities of daily living (IADL), Geriatric Depression Scale (GDS), and diabetes mellitus were considered as confounders.

Abbreviation: CI, confidence interval.

Table 4 presents the indirect effects of each mediator and its respective proportions. In men, significant indirect effects were observed for having dental checkups within 1 year, tooth brushing frequency of twice a day or more, not drinking alcohol, and not smoking. For women, a significant indirect effect was observed for not smoking. However, the indirect effects of tooth brushing frequency of twice a day or more and not drinking alcohol in women were not significant. Tooth brushing frequency of twice a day or more had the highest significant mediating proportion in men (22.8%) but this proportion was only 2.5% in women, which was not significant. Not smoking had the highest significant mediating effect on women (10.1%), and it was the second highest in men (10.0%). Not drinking alcohol had a 2.9% mediated proportion in men; however, a non‐significant negative mediated proportion (−0.5%) was observed in women.

**Table 4:** Proportion mediated by mediators between marital status and the number of teeth by the Karlson–Holm–Breen (KHB) method with multiple imputation (*n* = 21 745)

Category	Mediator	Indirect effect	Proportion mediated
		Coefficient (95% CI)	%
Men (*n* = 10 374)	Dental treatment within 1 year	0.029 (−0.004; 0.062)	2.0
	Dental checkup within 1 year	0.126 (0.065; 0.188)	8.9
	Tooth brushing frequency of twice a day or more	0.324 (0.228; 0.420)	22.8
	Not drinking alcohol	0.042 (0.008; 0.075)	2.9
	Not smoking	0.142 (0.067; 0.216)	10.0
Women (*n* = 11 371)	Dental treatment within 1 year	0.001 (−0.004; 0.008)	0.2
	Dental checkup within 1 year	0.010 (−0.033; 0.054)	1.3
	Tooth brushing frequency of twice a day or more	0.019 (−0.032; 0.071)	2.5
	Not drinking alcohol	−0.004 (−0.011; 0.003)	−0.5
	Not smoking	0.079 (0.045; 0.113)	10.1

Abbreviation: CI, confidence interval.

Supplementary Tables S2–S4 show the results of the sensitivity analysis. The results of the sensitivity analysis with detailed marital status categories demonstrated that the association of marital status with the number of teeth attenuated when comparing the confounders‐adjusted model, and mediators and confounders‐adjusted model. For men, the coefficients for bereavement changed from −1.12 (95% CI = −1.86; −0.36) in the confounders‐adjusted model to −0.69 (95% CI = −1.41; 0.02) in the mediators and confounders‐adjusted model. Similarly, the coefficients for being separated shifted from −2.49 (95% CI = −3.53; −1.45) to −1.57 (95% CI = −2.56; −0.58). For women, the coefficient for bereavement shifted from −0.98 (95% CI = −1.40; −0.55) to −0.92 (95% CI = −1.33; −0.51), while the coefficient of being separated shifted from −0.78 (95% CI = −1.53; −0.04) to −0.40 (95% CI = −1.14; 0.33).

## Discussion

This study provides valuable insights into the association between marital status and the number of teeth. Men and women with a spouse had 1.42 and 0.78 more natural teeth than those without a spouse, after controlling for sociodemographic and health‐related confounders (Table 2). Oral health‐related behaviors mediated this association by 46.62% and 13.68% for men and women, respectively. These results support the hypothesis that having a spouse has a positive association with oral health behaviors and the number of teeth.

Previous studies demonstrated an association between spouses' habits, particularly regarding smoking, alcohol consumption, and exercise.^32^ Similarly, marital status has been linked to comparable oral health behaviors among couples with young children owing to shared knowledge, attitudes, and practices,^15^ as well as to better access to dental care, with married individuals in middle or late adulthood being more likely to seek dental services than their unmarried counterparts.^16^ Our study adds to the literature showing that oral health behaviors mediate the association between marital status and the number of teeth in older adults. Our results also align with previous studies that showed that women tend to be more conscientious about maintaining oral health.^33^

Our mediation analysis explained the mechanisms mediating the association between marital status and the number of teeth. Oral health behavior‐related mediators exhibited a larger proportion of mediation in men than in women. The frequency of tooth brushing has emerged as a key mediator in older men. This result may be attributed to the prevalence of tooth brushing as the primary oral hygiene routine among older adults.^34^ Women brush their teeth more frequently than men,^35^ serving as positive role models and influencing their partners. Dental checkups had higher mediation effects in men than in women. Women were more likely to seek dental treatment.^36^ Wives who prioritize oral health may encourage their husbands to emphasize preventive care. In this study, the prevalence of smoking was similar to that reported in previous research, which suggests that having a spouse is associated with lower alcohol and tobacco use among men.^37^ However, men with a spouse in the present study exhibited higher alcohol consumption, which may have been influenced by social gatherings and cultural factors.^38^

Previous studies suggested the large impacts of social networks on oral health behaviors.^39^ Emotional well‐being may also play a crucial role in oral health and may help explain the relationship between marital status and the number of teeth.^40^ Stress contributes to oral health conditions, including periodontal diseases.^41^ Separation and bereavement, as marital transitions, often lead to acute stress and disruptions considered to have adverse effects on oral health.^42^ Having a supportive spouse can help mitigate stress, create an environment for better oral health practices, and ultimately, for a higher number of teeth in old age.^43^ A reciprocal relationship exists between social isolation and oral health in older adults.^44^ Our sensitivity analysis demonstrates gender differences in how marital status influences the number of teeth, particularly after adjusting for mediators and confounders. Overall, the association between marital status and the number of teeth was attenuated in all categories of marital status for men when oral health behaviors were considered. Among women, the attenuation was remarkable for those who separated. For bereaved men, the association with fewer teeth became non‐significant, suggesting mediation by oral health behaviors, whereas for bereaved women, the association remained significant, suggesting a weak mediation by oral health behaviors and the influence of other factors. For separated women, the association weakened and became non‐significant, suggesting mediation by oral health behaviors. Notably, never‐married women showed a positive association with a higher number of teeth. While married women are often involved with their husbands, unmarried older women need to interact with other people. For this reason, unmarried women may face strong social pressure to maintain good oral health. Future studies should consider stress and social relationships as mediators in the association.

The association between marital status and the number of teeth has important implications for dental public health. Encouraging couples to prioritize oral health together may lead to positive behavioral changes and improved outcomes. Special attention should be given to individuals without a spouse, ensuring that they receive the support needed for optimal oral health. Future research should explore how marital status impacts oral health through key factors such as shared health behaviors (e.g., oral hygiene and dental visits), social support (e.g., spousal encouragement and resource access), and psychological well‐being (e.g., stress reduction and emotional health). Understanding these pathways may provide deeper knowledge into the connection between marriage and oral health. Additionally, given gender differences, studies should explore the specific factors mediating these effects to develop gender‐sensitive oral‐health‐promotion strategies.

This study has both limitations and strengths. First, the cross‐sectional analysis did not indicate a causal relationship. A longitudinal study may reveal whether changes in marital status lead to changes in the number of teeth over time. Second, marital status represents only a single dimension of an individual's social network. Social networks are multifaceted and encompass various relationships and interactions (family, friends, community and social engagement, work, and professional networks) extending beyond marriage.^45^ Third, the number of natural teeth was assessed through self‐report rather than through clinical examination, which may introduce measurement errors. However, given the large sample size and feasibility constraints of conducting dental examinations, self‐reported data remains a practical and widely accepted method for assessing oral health in epidemiological studies, such as the JAGES study. Fourth, the 75 municipalities were not randomly selected. Therefore, the generalizability of the study results was limited.

The strength of this study is the comprehensive approach of considering multiple mediators to explore the association between marital status and the number of teeth. Second, this study specifically emphasizes gender distinctions in the analysis. By examining the associations for men and women, this study recognized potential variations in how marital status influenced oral health behaviors based on gender.

In conclusion, being married in old age offers a range of benefits that contribute to the maintenance of oral health, as shown in the results from the analyses controlling for confounding factors. Oral health‐related behaviors partially contributed to this association.

## Disclosure statement

The authors have no conflicts of interest to declare.

## Author contributions

Farzana Sharmin: Conceptualization, Methodology, Formal analysis, Software, Writing – original draft, Visualization. Jun Aida: Conceptualization, Methodology, Investigation, Funding acquisition, Resources, Writing – review and editing, Supervision. Yusuke Matsuyama: Investigation, Writing – review and editing. Shiho Kino: Investigation, Writing – review and editing. Sakura Kiuchi: Investigation, Writing – review and editing. Katsunori Kondo: Funding acquisition, Writing – review and editing. All authors provided their final approval and agreed to be accountable for all aspects of this study.

## Ethics statement

The Japan Gerontological Evaluation Study (JAGES) 2022 was reviewed and approved by the Ethics Committee on Research of Human Subjects at the Chiba University Graduate School of Medicine (No. M10460) and the Institute of Science Tokyo (No. D2022‐040). The study adhered to the Strengthening the Reporting of Observational Studies in Epidemiology (STROBE) guidelines for cross‐sectional studies.

## Supporting information


**DATA S1.** Supplementary Method: Detailed explanation of mediators and confounders.

**Table S1.** Frequency of missing responses for each variable
**Table S2:** Gender‐stratified descriptive statistics of marital status categories after multiple imputation (Men, *n* = 10 374)
**Table S3:** Gender‐stratified descriptive statistics of marital status categories after multiple imputation (Women, *n* = 11 371).
**Table S4:** Gender‐stratified linear regression analysis of the association of marital status categories and mediators with number of teeth after multiple imputation (*n* = 21 745)

## Data Availability

The data that support the findings of this study are available from the Japan Gerontological Evaluation Study (JAGES); however, restrictions apply to the availability of these data, which were used under license for the current study, and so are not publicly available. Data are, however, available from the authors upon reasonable request and with permission from JAGES.
