# Supplementary material for: Gender differences in roles of health behavior between marital status and oral health
Source: Geriatr Gerontol Int. 2025 Sep 11;25(10):1397–403. doi: 10.1111/ggi.70170 (PMC12501663; doi:10.1111/ggi.70170)
Supplement: Supplementary file 1 — DATA S1. Supplementary Method: Detailed explanation of mediators and confounders. [file GGI-25-1397-s001.docx]

Supplementary Method. Detailed explanation of mediators and confounders

Mediators

Dental treatment was assessed by the question: “When was the last time you visited a dentist for treatment (including adjustment of dentures)?” It was categorized as >1 year ago (1–3 years ago, >3 years ago, I have never visited a dentist) or within 1 year (within 6 months, 6 months to 1 year). Dental checkups were assessed by the question: “When was the last time you visited the dentist for other than treatment (e.g., checkups)?” It was categorized as > 1 year ago (1–3 years ago, >3 years ago, I have never visited a dentist) or within 1 year (within 6 months, 6 months to 1 year). Tooth brushing frequency was assessed by the question: “How often do you brush your teeth (including when someone else does it for you)?” It was categorized as once a day or less (once a day, sometimes, never brush) and twice a day or more (>3 times a day, twice a day). Additionally, smoking status was assessed by the question: “Do you smoke cigarettes (including heated tobacco products, electronic cigarettes, etc.)?” It was categorized as yes (I smoke almost every day, I smoke occasionally) or no (quit within 5 years and do not smoke, quit >5 years and do not smoke, never smoked before). Alcohol drinking was assessed by the question: “Do you drink alcohol?” It was categorized as yes (currently drinking) or no (stopped within 5 years and not drinking now, stopped drinking >5 years ago and am not drinking now, never drank). Favorable oral health behaviors (i.e., having dental treatment within 1 year, having dental checkups within 1 year, tooth brushing frequency twice a day or more, not drinking alcohol, and not smoking) were coded as 1, and unfavorable oral health behaviors (i.e., having dental treatment >1 year ago, having dental checkups >1 year ago, tooth brushing frequency once a day or less, drinking alcohol, and smoking) were coded as 0.

Confounders

Demographic confounders included age (65-69, 70-74, 75-79, 80-84, and ≥85 years) and gender (men or women). Socioeconomic confounders consisted of years of education (≤9 years, 10-12 years, and ≥13 years) and annual household income, including pension (Japanese yen in millions; low, (<2.0), mid (2.0-3.9), and high (≥4.0)). Physical and mental health-related confounders included diabetes mellitus (yes/no), depressive symptoms (Geriatric Depression Scale (GDS-15); no depressive symptoms, (<5), mild depressive symptoms (5-9) and severe depressive symptoms (≥10)), and having limitation in Instrumental Activities of Daily Living (IADL) (having limitation and no limitation).
