# Supplementary material for: Gender differences in roles of health behavior between marital status and oral health
Source: Geriatr Gerontol Int. 2025 Sep 11;25(10):1397–403. doi: 10.1111/ggi.70170 (PMC12501663; doi:10.1111/ggi.70170)
Supplement: Supplementary file 2 — Table S1. Frequency of missing responses for each variable Table S2: Gender‐stratified descriptive statistics of marital status categories after multiple imputation (Men, n = 10 374) Table S3: Gender‐stratified descriptive statistics of marital status categories after multiple imputation (Women, n = 11 371). Table S4: Gender‐stratified linear regression analysis of the association of marital status categories and mediators with number of teeth after multiple imputation (n = 21 745) [file GGI-25-1397-s002.docx]

**Supplementary Table 1: Frequency of missing responses for each variable**

|  | Missing |
| --- | --- |
| Variable | n (%) |
| Number of teeth | 2372 (10.9%) |
| Marital status | 236 (1.1%) |
| Sex | 0 (0.0%) |
| Age | 0 (0.0%) |
| Education level | 590 (2.7%) |
| Annual household income | 2872 (13.2%) |
| IADL | 1855 (8.5%) |
| GDS | 3156 (14.5%) |
| Diabetes Mellitus | 666 (3.1%) |
| Dental treatment | 905 (4.2%) |
| Dental checkup | 1091 (5.0%) |
| Tooth brushing | 767 (3.5%) |
| Alcohol drinking* | 890 (4.1%) |
| Smoking** | 399 (1.8%) |

Note: Data are presented as mean (SD) for continuous measures, and n (%) for categorical measures

Abbreviation: SD, Standard deviation; IADL, Instrumental activity of daily living; GDS, Geriatric depression scale.

*Alcohol Drinking: Yes (Currently drinking); No (Stopped within 5 years and not drinking now, stopped drinking > 5 years ago and am not drinking now, never drank to begin with)

**Smoking: Yes (I smoke almost every day, I smoke occasionally); No (Quit within 5 years and do not smoke now, quit > 5 years ago and do not smoke now, never smoked before)

**Supplementary Table 2: Gender-stratified descriptive statistics of marital status categories after multiple imputation (Men, n=10374).**

|  | **Men** | **Having a spouse** | **Bereavement** | **Separated** | **Never married** | **Other** |
| --- | --- | --- | --- | --- | --- | --- |
|  | **n = 10374** | **n = 8651** | **n = 788** | **n = 377** | **n = 459** | **n = 99** |
|  |  | % | % | % | % | % |
| **Number of teeth, mean (SD)** |  | 18.48 (9.60) | 15.96 (10.81) | 15.85 (10.36) | 18.08 (9.77) | 13.28 (11.31) |
| **Age** | 65-69 | 22.66 | 9.41 | 33.52 | 42.26 | 27.23 |
|  | 70-74 | 29.94 | 22.59 | 34.04 | 35.31 | 27.28 |
|  | 75-79 | 23.87 | 21.88 | 19.78 | 15.34 | 18.17 |
|  | 80-84 | 15.73 | 25.15 | 10.61 | 5.12 | 15.15 |
|  | ≥85 | 7.80 | 20.97 | 2.05 | 1.97 | 12.18 |
| **Education level** | ≤ 9 years | 18.34 | 26.18 | 23.27 | 20.32 | 34.27 |
|  | 10-12 years | 41.50 | 38.23 | 41.67 | 42.68 | 36.03 |
|  | ≥ 13 years | 40.16 | 35.59 | 35.06 | 37.00 | 29.69 |
| **Income (million JPY)** | Low (<2.0) | 44.74 | 42.01 | 58.10 | 65.01 | 70.96 |
|  | Mid (2.0-3.9) | 41.53 | 41.39 | 33.04 | 29.48 | 25.47 |
|  | High (≥4.0) | 13.73 | 16.60 | 8.86 | 5.51 | 3.57 |
| **IADL** | Limitation | 25.97 | 23.33 | 15.09 | 10.94 | 16.31 |
|  | No limitation | 74.03 | 76.67 | 84.91 | 89.06 | 83.69 |
| **GDS** | Non | 79.15 | 69.41 | 58.95 | 56.61 | 46.75 |
|  | Mild | 17.34 | 22.70 | 28.67 | 30.93 | 37.59 |
|  | Severe | 3.51 | 7.89 | 12.38 | 12.46 | 15.65 |
| **Diabetes mellitus** | Not present | 81.51 | 77.12 | 77.12 | 79.26 | 87.22 |
|  | Present | 18.49 | 22.88 | 22.88 | 20.74 | 12.78 |
| **Dental treatment** | More than 1 year ago | 40.21 | 43.61 | 53.00 | 51.81 | 59.64 |
|  | Within 1 year | 59.79 | 56.39 | 47.00 | 48.19 | 40.36 |
| **Dental checkup** | More than 1 year ago | 47.34 | 51.31 | 58.50 | 59.50 | 69.80 |
|  | Within 1 year | 52.66 | 48.69 | 41.50 | 40.50 | 30.20 |
| **Tooth brushing** | Once a day or less | 33.49 | 43.65 | 48.71 | 49.39 | 42.88 |
|  | Twice a day or more | 66.51 | 56.35 | 51.29 | 50.61 | 57.12 |
| **Alcohol Drinking*** | Yes | 62.28 | 57.50 | 59.40 | 52.47 | 54.91 |
|  | No | 37.72 | 42.50 | 40.60 | 47.53 | 45.09 |
| **Smoking**** | Yes | 16.12 | 15.45 | 28.47 | 25.20 | 23.65 |
|  | No | 83.88 | 84.55 | 71.53 | 74.80 | 76.35 |

Note: Descriptive statistics obtained after multiple imputation

Abbreviation: SD, Standard deviation; IADL, Instrumental activities of daily living; GDS, Geriatric depression scale

*Alcohol Drinking: Yes (currently drinking); No (stopped within 5 years and not drinking now, stopped drinking > 5 years ago and not drinking now, never drank to begin with)

**Smoking: Yes (I smoke almost every day, I smoke occasionally); No (Quit within 5 years and do not smoke now, quit > 5 years ago and do not smoke now, never smoked before)

**Supplementary Table 3: Gender-stratified descriptive statistics of marital status categories after multiple imputation (Women, n=11371).**

|  | **Women** | **Having a spouse** | **Bereavement** | **Separated** | **Never married** | **Other** |
| --- | --- | --- | --- | --- | --- | --- |
|  |  |  |  |  |  |  |
|  | **n= 11371** | **n= 7088** | **n= 3305** | **n= 603** | **n= 304** | **n= 71** |
|  |  | % | % | % | % | % |
| **Number of teeth, mean (SD)** |  | 20.07 (8.68) | 16.61 (10.47) | 18.78 (9.44) | 20.77 (8.52) | 16.24 (10.95) |
| **Age** | 65-69 | 28.99 | 9.32 | 31.51 | 30.26 | 14.08 |
|  | 70-74 | 33.94 | 20.85 | 33.90 | 32.89 | 18.31 |
|  | 75-79 | 22.10 | 24.90 | 21.40 | 20.07 | 23.94 |
|  | 80-84 | 11.96 | 26.23 | 9.50 | 10.86 | 30.99 |
|  | ≥85 | 3.01 | 18.70 | 3.70 | 5.92 | 12.68 |
| **Education level** | ≤ 9 years | 19.08 | 32.16 | 24.38 | 16.45 | 40.60 |
|  | 10-12 years | 47.60 | 45.12 | 46.27 | 44.08 | 29.00 |
|  | ≥ 13 years | 33.32 | 22.72 | 29.35 | 39.47 | 30.40 |
| **Income (million JPY)** | Low (<2.0) | 46.70 | 63.03 | 66.50 | 60.20 | 74.65 |
|  | Mid (2.0-3.9) | 41.14 | 28.65 | 27.70 | 32.80 | 18.31 |
|  | High (≥4.0) | 12.16 | 8.32 | 5.80 | 7.00 | 7.04 |
| **IADL** | Limitation | 40.70 | 36.31 | 22.45 | 31.25 | 30.99 |
|  | No limitation | 59.30 | 63.69 | 77.55 | 68.75 | 69.01 |
| **GDS** | Non | 77.27 | 73.65 | 67.50 | 73.03 | 61.97 |
|  | Mild | 18.48 | 20.60 | 23.55 | 20.07 | 29.58 |
|  | Severe | 4.25 | 5.75 | 8.95 | 6.90 | 8.45 |
| **Diabetes mellitus** | Not present | 89.73 | 88.71 | 89.55 | 90.46 | 88.73 |
|  | Present | 10.27 | 11.29 | 10.45 | 9.54 | 11.27 |
| **Dental treatment** | More than 1 year ago | 34.82 | 37.55 | 43.28 | 34.00 | 49.30 |
|  | Within 1 year | 65.18 | 62.45 | 56.72 | 66.00 | 50.70 |
| **Dental checkup** | More than 1 year ago | 38.8 | 42.00 | 46.60 | 38.16 | 50.70 |
|  | Within 1 year | 61.2 | 58.00 | 53.40 | 61.84 | 49.30 |
| **Tooth brushing** | Once a day or less | 12.51 | 16.79 | 15.26 | 11.51 | 22.54 |
|  | Twice a day or more | 87.49 | 83.21 | 84.74 | 88.49 | 77.46 |
| **Alcohol Drinking*** | Yes | 23.98 | 18.88 | 27.60 | 25.00 | 25.35 |
|  | No | 76.02 | 81.12 | 72.40 | 75.00 | 74.65 |
| **Smoking**** | Yes | 3.30 | 3.30 | 11.60 | 7.00 | 1.41 |
|  | No | 96.70 | 96.70 | 88.40 | 93.00 | 98.59 |

Note: Descriptive statistics obtained after multiple imputation

Abbreviation: SD, Standard deviation; IADL, Instrumental activities of daily living; GDS, Geriatric depression scale

*Alcohol Drinking: Yes (currently drinking); No (stopped within 5 years and not drinking now, stopped drinking > 5 years ago and not drinking now, never drank to begin with)

**Smoking: Yes (I smoke almost every day, I smoke occasionally); No (Quit within 5 years and do not smoke now, quit > 5 years ago and do not smoke now, never smoked before

**Supplementary Table 4: Gender-stratified linear regression analysis of the association of marital status categories and mediators with number of teeth after multiple imputation (n= 21745)**

|  |  | **Univariable Model** | **Confounders adjusted model** | **Mediators and Confounders adjusted model** |
| --- | --- | --- | --- | --- |
|  | | **Coefficient (95%CI)** | **Coefficient (95% CI)** | **Coefficient (95% CI)** |
| **Men (n =10374)** |  |  |  |  |
| **Marital status** | Having a spouse | Reference | Reference | Reference |
|  | Bereavement | -2.51 (-3.30; -1.73) | -1.12 (-1.86; -0.36) | -0.69 (-1.41; 0.02) |
|  | Separated | -2.63 (-3.70; -1.55) | -2.49 (-3.53; -1.45) | -1.57 (-2.56; -0.58) |
|  | Never married | -0.40 (-1.32; 0.512) | -0.58 (-1.47; 0.31) | 0.26 (-0.59; 1.13) |
|  | Other | -5.20 (-7.42; -2.97) | -3.78 (-5.86; -1.70) | -3.01 (-4.97; -1.06) |
| **Women (n =11371)** |  |  |  |  |
| **Marital status** | Having a spouse | Reference | Reference | Reference |
|  | Bereavement | -3.46 (-3.87; -3.04) | -0.98 (-1.40; -0.55) | -0.92 (-1.33; -0.51) |
|  | Separated | -1.28 (-2.07; -0.50) | -0.78 (-1.53; -0.04) | -0.40 (-1.14; 0.33) |
|  | Never married | 0.69 (-0.27; 1.67) | 0.92 (-0.01; 1.87) | 0.93 (.001; 1.86) |
|  | Other | -3.82 (-6.37; -1.28) | -1.32 (-3.75; 1.10) | -0.97 (-3.39; 1.43) |

Note: The Mediators and Confounders adjusted model included all listed variables simultaneously and adjusted for confounders, age, gender, education level, annual household income, instrumental activity of daily living (IADL), geriatric depression scale (GDS), diabetes mellitus and mediators (dental treatment, dental checkup, tooth brushing frequency, alcohol drinking and smoking). Confounders adjusted model included age, gender, education level, annual household income, instrumental activities of daily living (IADL), geriatric depression scale (GDS), and diabetes mellitus.

Abbreviation: SD, Standard deviation; IADL, Instrumental activity of daily living; GDS, Geriatric depression scale

*Alcohol Drinking: Yes (Currently drinking); No (Stopped within 5 years and not drinking now, stopped drinking > 5 years ago and not drinking now, never drank to begin with)

**Smoking: Yes (I smoke almost every day, I smoke occasionally); No (Quit within 5 years and do not smoke now, quit > 5 years ago and do not smoke now, never smoked before)
